# Meditation-Induced Psychosis: Trigger and Recurrence

**DOI:** 10.1155/2021/6615451

**Published:** 2021-08-13

**Authors:** Sulochana Joshi, Anusha Manandhar, Pawan Sharma

**Affiliations:** ^1^Department of Psychiatry, Patan Academy of Health Sciences, Lalitpur, Nepal; ^2^Department of Psychiatry, Patan Academy of Health Sciences, Lalitpur, Nepal; ^3^Department of Psychiatry, Patan Academy of Health Sciences, Lalitpur, Nepal

## Abstract

Meditation is regarded as a self-regulation approach to manage emotions. Meditation has a beneficial effect on mental health. Different kinds of meditation are practiced in many religions and cultures for the general wellbeing of an individual. However, meditation-related experiences and negative effects of meditation are not uncommon. Meditation-induced psychosis has been reported in the past. Here, we present a case of a 33-year-old male patient who developed acute and transient psychosis twice after meditation and discuss the role of meditation as a precipitating factor to psychosis.

## 1. Introduction

Meditation is generally used to denote various forms of mental exercises with techniques of concentration or contemplation [1]. It is a state between attention to an object and absorption within it [2]. Meditation is a technique in which an individual practices activity to achieve a mentally clear and emotionally calm and stable state. This is done either by mindfulness or focusing on an object or thoughts [3]. In Vipassana meditation, the meditator observes his own thoughts and sensations in a motionless seated position and meditates for hours [4]. Meditation is regarded as a self-regulation approach to reduce the stress and manage the emotion [5]. Meditation has a significant role in chronic illness such as hypertension, insomnia, and chronic pain [6]. Meditation generally has a beneficial effect on mental illnesses such as anxiety disorder, mood disorder, substance use disorder, and suicidality [7]. However, a few reports suggest that meditation can have hazards. As reviewed by West et al., meditation can induce serious psychological side effects, including depersonalization, derealization, and psychotic symptoms like hallucinations as well as mood disturbances [8]. There is also evidence that meditation may even lead to psychosis or worsen it in some cases [9]. In 1915, Morita was the first to describe psychotic symptoms related to meditation in superstitious people with low educational levels and considered the phenomena as a culture-specific disorder [10]. It has also been described as a culture-bound syndrome [11] .

Here, we present a case from our tertiary care center with recurrence of psychosis after practicing meditation. There are some previous case reports of psychotic disorder having association with meditation, but it has been difficult to show cause effect relationship between the two. This paper reports a case in which psychosis was precipitated by intensive meditation (Vipassana) twice with an attempt to show the temporal relation between psychosis and meditation.

## 2. Case

The case was a 33-year-old married male of middle socioeconomic status, who worked as a part-time officer in a private office and was preparing for a government officer job. He had a well-adjusted premorbid personality with culturally accepted religious views. There was no history of any medical problems, trauma, or substance use as well as no history of any psychiatric illness in the family. The patient presented to the emergency department (ED) with abnormal behaviour and difficulties to be at home because of suspiciousness, aggressive behaviour, and disturbed sleep for five days. The family members had tried to calm him down with the psychotropic risperidone 2 mg given from a nearby health centre for two days ahead of the presentation at ED.

On further evaluation, the family members gave a history of similar symptoms nine years earlier following ten days of intensive Vipassana meditation. At that time, the patient had a history suggestive of delusions of persecution and reference, auditory hallucination 2^nd^ person commenting type, and decreased sleep. His symptoms had completely remitted within one month of taking psychotropic medication, details of which is not known.

On detailed evaluation of the current episode of the patient, family members revealed that apart from the episode nine years back, he never had any other such episodes. However, thinking no harm can occur after many years, the patient had gone for a 20-day meditation program in the Vipassana meditation centre for a second time. He had been practicing the same intensive meditation for 18 days as in the past prior to the onset of illness. In this Vipassana meditation, the patient along with other participants would practice meditation for about 18 hours a day, eat once a day, and sleep as little as possible, i.e., four to five hours a night. During the Vipassana meditation, he had to sit in squatting position with eyes closed taking natural breaths in the meditation hall, a quiet place. He had to focus his full attention to his body movements and sensations. He should not communicate with anyone by any means throughout the stay, nor involve in any other activity apart from meditation.

Everything was going smoothly until the 16^th^ day of intensive meditation, when the patient started saying people were looking at him and trying to harm him, even though no one noticed any such activity. Since the patient was found self-muttering, aggressive towards other participants, and not meditating, he was sent home on the 18^th^ day. At home also, he repeatedly expressed of harm from other unknown people, was firm about it, and did not believe his friends and family when they told there was no such activity. While awake and in full consciousness, he also started expressing of hearing voices—with his both ears—clearly coming from the room next to. These voices were not heard by others. The patient was restless and aggressive and frequently expressed that he could hear voices saying family members were trying to harm him. He accused his wife of having an extramarital affair with his brother in his absence. He tore her clothes and burnt them and verbally abused his wife if she enquired about doing so. He blamed his wife and other unknown people conspiring against him and wanting to kill him by poisoning his food. He threw the food when served and physically assaulted his wife claiming the food is poisoned. The family members attempt to reassure him, explaining to him that none of his suspicions was true and that they did not hear any voices of harming him went in vain as the patient did not believe them. During this time, he slept for only two to three hours, eat less amount of food than usual, did not perform his usual self-care such as brushing his teeth or bathing, stopped going to work, and did not interact with family members resulting into marked sociooccupational dysfunction. After observing him for a day at home, the family members then had taken the patient to the local health centre, where he was prescribed risperidone 2 mg at night. The patient's symptoms continued despite giving risperidone 2 mg daily for two days, and it was not possible to contain him at home. Therefore, the family members brought the patient to ED where psychiatric consultation was done.

In the mental state examination, the patient was irritable and had increased psychomotor activity, delusions of persecution and infidelity, 2^nd^ person auditory hallucination, absent insight, and rapport was not established. With a diagnosis of acute and transient psychotic disorder (ATPD) according to International classifications of diseases, tenth version (ICD-10), the patient was then admitted in the psychiatry ward [12].

In the ward, the patient continued expressing suspiciousness and was irritable. On day 1, Brief Psychiatric Rating Scale (BPRS) score was 57 and Clinical Global Impression-Severity Scale (CGI-S) score was 6 [13, 14].

On day 3, the patient's aggression was mild towards the family members and decreased on subsequent stay in the ward. He still was suspicious towards the wife for cheating on him but stopped reporting on hearing voices unheard to others. BPRS score was 44; CGI-S was 5.

The patient was managed with oral risperidone 2 mg daily initially for two days, then was optimized to 6 mg per day in two divided doses after one week and in addition clonazepam 0.25 mg morning and evening. The clonazepam dose was tapered over one week when the patient's biorhythm was normal and psychotic symptom was absent. The patient was discharged on 10^th^ day of hospital admission. At the time of discharge, his sleep, appetite, and self-care was normal, the psychotic symptoms had remitted, and BPRS and CGI-S scores were 19 and 2, respectively. He was compliant to medication, and insight was present.

The patient was advised to continue with risperidone and not to practice meditation further. Patient was asked to follow up after two weeks. The patient was maintaining well on his first follow-up. He was then asked to a second follow-up in the next one month, wherein the patient was doing well too without any signs of extrapyramidal side effects. The patient was advised to follow up in the next 3 months and psychoeducated about the need of continuation of risperidone in maintenance dosage at least for a year.

## 3. Discussion

This case documents an association of psychotic symptoms with intensive meditation. The patient was maintaining well without medication for about nine years after a first episode of acute psychosis, and then, a second psychotic episode recurred on 16th day of practicing similar intensive meditation. The patient described in this case report experienced psychotic symptoms precipitated by intensive meditation in both episodes of illness and without any symptoms between the two episodes, suggesting a temporal association between meditation and psychosis. According to the Naranjo algorithm, it also indicates a ‘possible' association between meditation practice and psychotic symptoms in this case [15]. There is also evidence suggesting that meditation alone is not the only causal factor for psychosis, but other factors like sleep and sensory deprivation and fasting during meditation may precipitate psychotic episodes [6]. In our case, various components of meditation like sleep deprivation, fasting, social isolation, long hours of meditation, and a past history of psychotic experiences could have been contributing factors. There are case reports suggesting meditation could be a precipitating factor for psychosis [16]. As underlined by a review about Qi-gong-induced mental disorders, psychotic deterioration is the result of meditation that acts as a stressor in vulnerable individuals [17]. Of the proposed neurobiological factors behind this association are increased levels of neurotransmitters like dopamine, serotonin, and acetylcholine [18] during meditation. There is also evidence of increased cerebral blood flow in specific brain areas like the frontal lobe, prefrontal cortex (PFC), thalamus, hippocampus, hypothalamus, and cingulate gyrus during meditation [19, 20]. These findings and the findings on these areas in the patients with schizophrenia regarding synaptic connectivity and neuronal thickness of PFC could have some relation to the pathophysiology of psychosis [21]. However, risk of serious mental illness being precipitated by meditation is less recognized.

Attending meditation is common and is a regular practice in different parts of the world in different forms for wellbeing as well as religious purposes. When meditation is practiced under proper guidance and in moderation, meditation can enhance psychological wellbeing [22]. Even individuals with psychosis can benefit if the practice of meditation is tailored accordingly [23]. Further research is required to delineate the phenomenology of psychosis induced by meditation practices. Also, longitudinal research methodology with adequate sample size is needed to exactly know the associations of meditation and psychosis.

## 4. Conclusion

There is a significant role of meditation in treatment of chronic somatic illnesses as well as mental disorders. However, the practice of meditation should be adjusted according to each meditator's need and monitored throughout by the meditation teachers. For the benefit of the meditators, some form of collaboration between mental health experts and meditation teachers is needed. There are some case reports of psychotic disorder having association with meditation, but it is difficult to show a cause-effect relationship between the two. This case report demonstrates the possibility of association between meditation and psychosis as well as meditation as the stressor for causation of psychosis. There is a need of longitudinal in-depth research to delineate the association between meditation and psychosis in general and the role of different types of meditation and meditators.

## Data Availability

The authors confirm that all the information regarding the patient can be found within the article.
